# Comorbidity in spinal cord injury in Iran: A narrative review

**DOI:** 10.1515/tnsci-2022-0343

**Published:** 2024-07-05

**Authors:** Taher Taheri, Saereh Hosseindoost, Hadi Kazemi, Seyedehalia Kamali, Pirhossein Kolivand, Zeinab Gharaylou

**Affiliations:** Shefa Neuroscience Research Center, Khatamolanbia Hospital, Tehran, Iran; Brain and Spinal Cord Injury Research Center, Neuroscience Institute, Tehran University of Medical Sciences, Tehran, Iran; Pain Research Center, Neuroscience Institute, Imam Khomeini Hospital Complex, Tehran University of Medical Sciences, Tehran, Iran

**Keywords:** spinal cord injury, morbidity, Iranian patients

## Abstract

Spinal cord injury (SCI) is a severe medical condition that affects millions of people worldwide each year. In Iran, an estimated 9 out of every 100,000 individuals experience traumatic SCI occurrences. Long-term disabilities and comorbidities stemming from SCI often necessitate multiple therapeutic interventions. The aim of this study is to evaluate the morbidity in Iranian SCI patients. In this study, a four-step process was used to select, extract, analyze, and synthesize relevant literature. The search covered 750 records from five databases, resulting in 25 articles included in the review. These articles, published between 2000 and 2023, utilized cross-sectional, qualitative, or cohort designs. The findings explored the prevalence, risk factors, and consequences of comorbidities associated with SCI, categorized into four themes: physical, sexual, psychological, and metabolic morbidity. Physical morbidity refers to medical conditions or complications affecting body functions or structures in SCI patients. The most frequently reported cases include pressure ulcers, pain, osteoporosis, fractures, impaired pulmonary function, renal failure, and obesity. Metabolic morbidity includes conditions such as vitamin D deficiency and cardiometabolic risk factors. Psychological morbidity encompasses depression, anxiety, and adjustment disorders. Sexual morbidity refers to conditions or complications affecting the sexual function or satisfaction of SCI patients. This narrative literature review offers a comprehensive examination of various aspects of SCI in Iranian patients. The review identifies numerous challenges and difficulties faced by SCI patients while also highlighting protective factors that can improve their well-being. Additionally, the review acknowledges gaps and limitations within the current literature and suggests possible avenues for future research.

## 1 Introduction

Spinal cord injury (SCI) is a severe condition that results from damage to the spinal cord and associated nerves, leading to various sensory, motor, and autonomic dysfunctions. The causes of SCI can be categorized into two groups: traumatic and non-traumatic. Traumatic causes include accidents, falls, and violence, while non-traumatic causes consist of infections, tumors, and degenerative diseases [1]. The World Health Organization reports that SCI affects 13–33 million people globally, with 250,000–500,000 new cases each year [2]. SCI profoundly influences an individual’s physical, psychological, and social well-being as well as their families, healthcare systems, and society. The incidence of traumatic SCI in Iran is estimated to be 9 per 100,000. The prevalence of SCI in Iran, according to PubMed research, is 318.45 per million [3]. Additionally, the incidence and pattern of traumatic spine injury in a trauma center in southern Iran revealed a spine injury rate of 17.0% and a mortality rate of 15.5% [4]. These statistics underscore the significant impact of SCI on a global and national scale, emphasizing the need for continued research and effective interventions to address this public health concern.

SCI comorbidities often lead to long-term disabilities that require multiple treatments. Morbidity refers to health conditions such as diseases or disabilities that affect an individual’s health status and quality of life [5]. There are two classifications of morbidity in SCI patients: the primary and secondary types. Primary morbidity results from the direct consequences of SCIs on the nervous system, such as paralysis, neuropathic pain, and bladder and bowel dysfunctions. On the other hand, secondary morbidity refers to the indirect effects of SCIs on other body systems, such as pressure ulcers, infections, cardiovascular diseases, osteoporosis, and depression. Several factors affect morbidity in SCI patients, including the level and completeness of injury, time since injury, the patient’s age and gender, availability, and access to healthcare, and socio-cultural contexts [5]. These factors contribute to the development and management of comorbidities and secondary health conditions in SCI patients, impacting their overall health and well-being.

This review aims to achieve four objectives, which include describing various morbidities’ prevalence, risk factors, correlation, and consequences in SCI patients in Iran. Moreover, the review compares and contrasts research findings conducted in Iran, identifies strengths and weaknesses of the current literature, and provides implications and recommendations for education, clinical practice, policy-making, and research. The inclusion and exclusion criteria are explained in Section 2, and only up-to-date studies published in English or Persian from 2000 to 2023 were reviewed, primarily focusing on morbidity in SCI patients in Iran.

## 2 Methods

This narrative review followed a four-step process to select, extract, analyze, and synthesize the relevant literature on morbidity in SCI patients in Iran. The steps were as follows: we conducted a comprehensive literature search in five electronic databases: PubMed, Scopus, Science Direct, Semantic Scholar, and SID (Scientific Information Database).

In particular, the “AND” operator was employed to connect keywords and MeSH terms related to morbidity (such as morbidity, complications, comorbidity, secondary condition, and disability) and SCI (such as SCI, spinal cord trauma, paraplegia, and tetraplegia). These two concepts were simultaneously addressed in search results, enhancing their specificity. As part of the review’s focus, the “AND” operator was also utilized to restrict the search to studies conducted in Iran. The “OR” operator was employed to incorporate synonyms and variations into the search terms without compromising specificity. This approach allows for a thorough examination of relevant literature, regardless of linguistic nuances.

We applied the following inclusion criteria: (1) published in English or Persian; (2) published between 2000 and 2023; (3) focused on morbidity in SCI patients in Iran; and (4) used a quantitative or qualitative design. We excluded studies that (1) were not original research articles (e.g., reviews, editorials, and letters) and (2) did not report data on morbidity in SCI patients in Iran. The literature search was performed in February 2023 and updated in May 2023. To determine eligibility, we screened the titles and abstracts of the retrieved records using the inclusion and exclusion criteria. We obtained the full texts of the potentially eligible articles and assessed them for eligibility. Additionally, we checked the reference lists of the included articles for additional relevant studies. Any disagreements or uncertainties were resolved by discussion or consultation with a third reviewer.

The following data were extracted from each included article using a standardized data extraction form: (1) author(s), (2) year of publication, (3) title, (4) journal, (5) study design, (6) sample size, (7) mean age, (8) male percentage, (9) comorbidity percentage, (10) type of morbidity, (11) prevalence of morbidity, (12) risk factors of morbidity, and (13) consequences of morbidity. We also assessed the quality of each article using appropriate quality assessment tools based on the study design. For quantitative studies, we used the Joanna Briggs Institute Critical Appraisal Checklist for Analytical Cross-Sectional Studies. For qualitative studies, we used the Critical Appraisal Skills Programmed Qualitative Checklist. We rated each article as high, moderate, or low quality based on the number and severity of methodological flaws.

The literature search yielded a total of 750 records from the five databases. After removing duplicates and screening titles and abstracts, 25 articles met the inclusion criteria and were included in the review. The characteristics and quality ratings of the included articles are presented in Table 1. The articles used either a cross-sectional (*n* = 19), qualitative (*n* = 3), or cohort (*n* = 3) design. The quality ratings were high (*n* = 6), moderate (*n* = 17), or low (*n* = 2).

**Table 1:** Characteristics and quality ratings of the included articles

No.	Year	Journal	Design	Quality score	Quality rating	References
1	2023	Journal of Tissue Viability	Cross-sectional	9/12	Moderate	[6]
2	2019	Sexual Medicine	Qualitative	8/10	High	[7]
3	2018	Archives of Neuroscience	Qualitative	7/10	Moderate	[8]
4	2018	Archives of Physical Medicine and Rehabilitation	Qualitative	7/10	Moderate	[9]
5	2017	Spinal Cord	Descriptive cross-sectional	8/10	High	[10]
6	2017	Journal of Spinal Cord Medicine	Cross-sectional	9/12	Moderate	[11]
7	2017	Spinal Cord	Cross-sectional	8/12	Moderate	[12]
8	2016	American Journal of Men’s Health	Cross-sectional	6/10	Low	[13]
9	2015	The Journal of Spinal Cord Medicine	Cross-sectional	8/12	Moderate	[14]
10	2015	Journal of Spinal Cord Medicine	Analytical cross-sectional	8/10	High	[15]
11	2015	Topics in Spinal Cord Injury Rehabilitation	Cross-sectional	8/12	Moderate	[16]
12	2015	Spinal Cord	Cross-sectional	10/12	High	[17]
13	2014	Journal of Sleep Disorders and Therapy	Cross-sectional	7/10	Moderate	[18]
14	2014	Journal of Research in Medical Sciences	Cross-sectional	8/12	Moderate	[19]
15	2014	Advances in Clinical and Experimental Medicine	Retrospective cohort	8/10	High	[20]
16	2014	Iranian Journal of War and Public Health	Cross-sectional	5/12	Low	[21]
17	2014	Journal of Spinal Cord Medicine	Cross-sectional	7/10	Moderate	[22]
18	2014	Spinal Cord	Cross-sectional	9/12	Moderate	[23]
19	2014	The Journal of Spinal Cord Medicine	Cross-sectional	8/12	Moderate	[24]
20	2013	Spinal Cord	Cross-sectional	8/12	Moderate	[25]
21	2010	BMC Public Health	Cross-sectional	10/12	High	[26]
22	2009	Experimental and Clinical Transplantation	Cohort	8/11	Moderate	[27]
23	2009	Journal of Neurosurgery: Spine	Cross-sectional	9/12	Moderate	[28]
24	2006	Journal of Spinal Disorders and Techniques	Cohort	8/11	Moderate	[29]
25	2003	Spinal Cord	Cross-sectional	8/12	Moderate	[30]

## 3 Results

This narrative literature review is derived from 25 articles that meet the inclusion criteria. The studies shown in Table 1 were published between 2003 and 2023, with most appearing in the last decade. They come from various respected journals focusing on SCI, physical medicine, rehabilitation, and sexual health. Quality assessments, based on predetermined criteria, varied from low (*n* = 2) to high (*n* = 6), with most falling into the moderate quality category (*n* = 16).

### 3.1 Comorbidity in SCI

The articles outlined in Table 2 provide insights into the prevalence, risk factors, and implications of various comorbidities in individuals with SCI. These studies encompass a sample size that varies from 21 to 5,901, with the average age of participants ranging from 29.57 to 49.62 years. The proportion of male participants in these studies fluctuates between 0 and 100%, and the observed prevalence of comorbidities spans from 15 to 77%. The articles were categorized into four themes based on the type of morbidity: physical, sexual, psychological, and metabolic.

**Table 2:** Characteristics of the included articles based on comorbidity

No.	Sample size	Mean age	Male %	Type of morbidity	Prevalence of morbidity %	Risk factors of morbidity	Consequences of morbidity	References
1	2785	N/A	N/A	Pressure ulcers	16.1 for the SCI subgroup	Marital status, having SCI, urinary incontinence, level of education, treating center, number of days in ICU, age, and Glasgow coma scale score	Increased morbidity, mortality, and cost of care	[6]
2	60	38.3 men; 35.8 women	50	Sexual dysfunction	N/A	Injury level, injury duration, age, gender, and marital status	Reduced sexual function, intimacy, and well-being	[7]
3	51	35.8	84.3	Depressed mood	74	Gender, education, lesion type, and duration since the injury	Reduced quality of life, increased pain, and anxiety	[8]
4	31	N/A	0	Sexual dysfunction	N/A	Spinal injury type and level, lack of sexual knowledge and awareness post-SCI, and lack of sexual and reproductive education/counseling in the rehabilitation process	Reduced sexual quality of life and satisfaction	[9]
5	334	34.9	81.4	Pain (nociceptive or neuropathic)	50.7	Economic situation	Lower quality of life, depression, anger, poor adjustment, anxiety and sleep, and mood disorders	[10]
6	114	29.57	84.3	Adjustment disorder	28	N/A	Reduced rehabilitation, quality of life, and increased morbidity and mortality	[11]
7	319	34.6	100	Androgen deficiency and erectile dysfunction	29.1 androgen deficiency; 32.6 low total testosterone level	Injury level, injury duration, age, gender, and opioid use	Reduced sexual function and well-being	[12]
8	100	36.6	100	Impaired sexual function, such as erectile dysfunction, ejaculation dysfunction, orgasm dysfunction, and sexual satisfaction dysfunction	N/A	Injury level, age, marital status, education level, employment status, income level, duration of injury, medication use	Reduced quality of life, impaired sexual health, and intimacy	[13]
9		33.44	53.3	Passive knee stiffness and viscosity	N/A	N/A	SCI had significantly greater joint stiffness compared to able-bodied subjects	[14]
10	850	36.5	98	Obesity	42	Age, BMI	Increased risk of cardiovascular diseases, reduced functional level, increased risk of injury during nursing care	[15]
11	100	38.8	87	Poor sleep quality	77	Injury level, pain, spasticity, depression, anxiety, medication use, smoking, alcohol consumption, caffeine intake, physical activity, environmental factors	Reduced quality of life, impaired cognitive function, increased mortality risk, cardiovascular diseases, metabolic disorders, immune dysfunction, psychiatric disorders	[16]
12	60	34.6 SCI; 33.9 control group	100	Depressive mood and fatigue	26.7 and 40 for the SCI group	Injury level, injury duration, age, gender, and pain	Reduced physical and mental function and well-being	[17]
13	213	34.8	67.4	Depression and anxiety	15 Depression; 30 anxiety	Gender, education, employment, negative religious coping, and existential spiritual well-being	Reduced recovery, quality of life, and increased morbidity and mortality	[18]
14	160	38.8	81.9	Vitamin D deficiency	53.1	N/A	Calcium and vitamin D insufficiency in Iranian patients with SCI	[19]
15	200	49.9	100	Medical complications, such as urinary tract infection, pressure ulcer, osteoporosis, fracture, spasticity, and pain	N/A	Injury level, age, duration of injury, smoking status, BMI, marital status, education level, employment status	Increased mortality rate, decreased quality of life, and increased healthcare costs	[20]
16	368	49.62	100	Pulmonary function	N/A	Injury level, injury duration, age, gender, and smoking status	Reduced respiratory function and health status	[21]
17	100	38.8	87	Poor psychosocial outcomes such as depression, anxiety, stress, coping strategies, social support, and life satisfaction	N/A	Injury level, age, gender, marital status, education level, employment status, income level, duration of injury	Reduced quality of life, impaired mental health, increased suicide risk	[22]
18	105	41.0 SCI; 33.9 control group	0	Sexual dysfunction	88	Injury level, injury duration, age, education, symptoms of depression, and anxiety	Reduced sexual function and satisfaction	[23]
19	148	51	78.4	BMD loss in the spine and hip	N/A	Age, sex, BMI, injury completeness, injury level, ASIA score	Serum calcium, phosphor, and vitamin D levels,osteoporosis, and pathological fractures	[24]
20	162	34.6	100	Cardiometabolic risk factors, such as diabetes mellitus, dyslipidemia, hypertension, obesity, and smoking	N/A	16.7 diabetes mellitus, 22.2 dyslipidemia, 2.5 hypertension, 13.6 obesity, and 8 smoking	Increased risk of coronary heart disease, stroke, and mortality	[25]
21	123 (39 veterans: 84 non-veteran)	46.8 veterans: 41.4 non-veterans	100	Health-related quality of life measured by SF-36 questionnaire	N/A	Veteran status, age, marital status, education level, employment status, and income level	Reduced well-being and satisfaction	[26]
22	21	43.8	100	Renal failure	N/A	SCI status, age, sex, injury level, injury duration, and time since injury	Reduced risk of urolithiasis and upper urinary tract infection; increased risk of graft rejection	[27]
23	5901 (3791 traumatic, 2110 nontraumatic)	37.8 traumatic; 48.8 women	68	Pressure ulcer	39.2 (all), 71.8 (traumatic), 28.2 (nontraumatic)	Traumatic cause, older age, interval less than 1 year since the onset of SCI, male sex, single status, and lower education level	Negative impact on quality of life, rehabilitation set-backs, hospitalization, and death	[28]
24	100	38	100	Osteoporosis	N/A	SCI duration, level and completeness of injury, immobilization, hormonal changes, nutritional deficiencies, inflammation, oxidative stress, and altered bone metabolism	Increased risk of fragility fractures, infections, pain, and disability	[29]
25	169	N/A	100	Epididymo-orchitis	38.5	N/A	Azoospermia and infertility in paraplegic patients	[30]

#### 3.1.1 Physical morbidity

Physical morbidity refers to any medical condition or complication that affects the body functions or structures of people with SCI. The most common physical morbidities reported in this literature were pressure ulcers, pain, osteoporosis, fracture, pulmonary function impairment, renal failure, and obesity.

Pressure ulcers were the most prevalent physical morbidity among people with SCI, affecting more than half of the traumatic SCI subgroup and more than one-third of all SCI patients. Pressure ulcers were associated with increased morbidity, mortality, and cost of care. Risk factors for pressure ulcers include marital status, having SCI, urinary incontinence, level of education, treating center, number of days in ICU, age, Glasgow coma scale score, traumatic cause, interval less than 1 year since onset of SCI, male sex, and lower education level [6]. Other studies have identified additional risk factors such as incontinence, smoking, hypoalbuminemia, drinking alcohol, diabetes [31], immobility, vascular disease, impaired nutrition, perfusion issues, mechanical ventilation, and surgery [32]. Proper implementation of monitoring, education, and care programs can help reduce the occurrence of these ulcers [33].

Pain is another common physical morbidity among people with SCI. It can be classified into nociceptive or neuropathic pain, depending on the source and mechanism of pain generation. Pain is associated with lower quality of life, depression, anger, and poor adjustment, as well as anxiety, sleep, and mood disorders. The only risk factor for pain identified in one study was economic situation [10], which has been linked to pain intensity and pain-related factors such as pain interference and depressive symptoms among individuals with SCI [34]. Neuropathic pain is generally regarded as the most frequent type of pain after SCI [35].

Osteoporosis is a frequent physical morbidity among people with SCI that results from bone mineral density (BMD) loss in the spine and hip regions due to reduced mechanical loading on bones after SCI [29]. Osteoporosis increases the risk of fractures, which could lead to infections, pain, disability, and mortality [24,29]. Risk factors for osteoporosis include age, gender, body mass index (BMI), injury completeness and level and Asia Impairment Scale score, serum calcium levels, SCI duration level, immobilization, hormonal changes, nutritional deficiencies, inflammation, and oxidative stress altered bone metabolism [24].

Pulmonary function impairment is a physical morbidity among people with SCI that affects respiratory function and health status. It is caused by reduced lung volumes, impaired cough reflex, decreased chest wall compliance, and increased airway resistance. Risk factors for pulmonary function impairment include injury level and duration, as well as age, gender, and smoking [21].

Renal failure is a rare but serious physical morbidity among people with SCI who may require renal transplantation as a treatment option. Renal failure is caused by chronic kidney disease due to neurogenic bladder dysfunction, urolithiasis, and urinary tract infection. Renal transplantation reduces the risk of urolithiasis and upper urinary tract infection but increases the risk of graft rejection [27].

Obesity is a prevalent physical morbidity among people with SCI that affects more than one-tenth of the sample in one study. Obesity is defined as having a BMI greater than 30 kg/m^2^. Obesity increases the risk of cardiovascular diseases, reduced functional level, and increased risk of injury during nursing care. Risk factors for obesity include age and BMI [15].

#### 3.1.2 Sexual morbidity

Sexual morbidity refers to any condition or complication that affects the sexual function or satisfaction of people with SCI. Sexuality in individuals with SCI is significantly intricate and encounters clinical, social, and cultural obstacles [7,9,12,13,23].

#### 3.1.3 Psychological morbidity

##### 3.1.3.1 Depression

Depression is a common psychological morbidity reported in SCI patients. The prevalence of depression in SCI patients in Iran ranges from 15 to 74%, depending on the study. Depression is associated with several factors, such as age, gender, level and completeness of injury, time since injury, pain, anxiety, quality of life, and functional status. Depression has negative consequences on physical, psychological, and social well-being, such as reduced motivation, self-esteem, coping skills, social support, and sexual satisfaction. Pain is one of the consistent and significant risk factors for depression in SCI patients in Iran. Other risk factors for depression include anxiety, low education, unemployment, low income, low self-esteem, low self-efficacy, low life satisfaction, low social support, and negative religious coping. Depression has a negative impact on SCI patients’ quality of life across all domains [8,16,17,18,22].

##### 3.1.3.2 Anxiety

Anxiety is another psychological morbidity among SCI patients. Anxiety is associated with lower quality of life, increased pain and depression, impaired sleep, and cognitive function. Risk factors for anxiety include gender education, employment, negative religious coping, and existential spiritual well-being [17,18,22]. A study from Iran found that 30% of the SCI patients had anxiety [17]. The findings indicated that religion and spiritual well-being have a moderating role in the occurrence of anxiety [37].

##### 3.1.3.3 Defense mechanisms

One study found that individuals with SCI who have adjustment disorder, a condition characterized by emotional distress and difficulty adapting to a changed situation, used more immature defense mechanisms such as denial, projection, and acting out than those without adjustment disorder. On the other hand, individuals with SCI who used more mature defense mechanisms such as humor, sublimation, and altruism had higher levels of self-esteem and lower levels of depression. The most prevalent defense style was neurotic, and the dominant defense mechanism used was idealization. The role of demographic and injury-related variables in determining the used defense mechanisms was found to be insignificant [11].

#### 3.1.4 Metabolic morbidity

Vitamin D deficiency is a prevalent metabolic morbidity among people with SCI that affects more than half of the sample in one study. Vitamin D deficiency is defined as having a serum 25-hydroxyvitamin D level less than 20 ng/ml. Vitamin D deficiency is associated with calcium and vitamin D insufficiency in Iranian patients with SCI [19].

Cardiometabolic risk factors are common metabolic morbidities among people with SCI that affect various aspects of the metabolic and cardiovascular system, such as glucose, lipid, blood pressure, and body weight. Cardiometabolic risk factors are associated with an increased risk of coronary heart disease, stroke, and mortality. Risk factors for cardiometabolic risk factors include diabetes mellitus, dyslipidemia, hypertension, obesity, and smoking [25]. Recent studies have suggested that vitamin D deficiency is associated with cardiometabolic risk factors, including obesity, autoimmune diseases, cancer, and insulin resistance [36].

## 4 Discussion

The results of this narrative review offer insights into the challenges faced by Iranians with SCIs. We gathered valuable data on the morbidity type, contributing factors and outcomes of various comorbidities affecting SCI patients in this population through the synthesis of the studies included. The occurrence of secondary conditions differs significantly based on the population studied and the research methods used. The results of the study showed that physical health problems secondary to SCI are extremely common worldwide [38]. SCI patients reported lower quality of life, higher levels of depression [8,16,17,18,22], anxiety [17,18,22], pain [10], sexual dysfunction [7,9,12,13,23], and osteoporosis [29]. These problems were influenced by various factors such as age, gender, level and duration of injury, marital status, education level, employment status, income level, access to health care services, social support, coping strategies, and spirituality. According to a study, among individuals with SCI for more than a decade, the most common secondary health issues were musculoskeletal pain, edema, neuropathic pain, and urinary tract infections, each with varying prevalence rates [39]. However, the evolution of these conditions over time has been explored in few longitudinal studies [40].

One of the key clinical implications of our findings is the high prevalence of pressure ulcers among SCI patients in Iran [6]. The findings of a comprehensive systematic review and meta-analysis indicate that pressure ulcers affect approximately one-third of patients with SCIs, suggesting a significant global prevalence [41]. This debilitating complication significantly impacts patient morbidity, mortality, and healthcare costs. Risk factors identified in the literature, such as urinary incontinence, immobility, and lower education level. The study examines a range of complex risk factors, including sensory impairment, immobility, and skin changes, as well as broader psychological and social factors [42]. These data underscore the urgent need for context-specific preventive measures to alleviate the burden of pressure ulcers in this patient population.

Vitamin D deficiency is a major health concern for people with SCI [43], affecting over 50% of the SCI population and influencing musculoskeletal [44,45] and cardiometabolic health [46]. A correlation has been reported between the occurrence of pressure ulcers and the vitamin D status in individuals with para- or tetraplegia [43]. Developing precise nutritional recommendations and supplementation strategies for this group is crucial to mitigating the chances of vitamin D deficiency and enhancing general health.

The synthesis of studies underscores the complex interplay between psychosocial factors and depression post-SCI, emphasizing the protective role of life satisfaction and social support and the need for targeted interventions addressing pain and community participation [47]. These mental health issues not only exacerbate physical symptoms but also have profound implications for overall well-being and quality of life [7,16,22,48]. Interventions aimed at addressing these psychological morbidities, such as cognitive-behavioral therapy and psychosocial support programs, are essential components of comprehensive SCI care.

The review of existing literature on SCI in Iran identified gaps in the research, including a lack of longitudinal or experimental studies, standardized instruments for measuring psychosocial outcomes, diversity in patient samples, and comparison groups. Future research should address these limitations by using more rigorous designs, methods, and samples, as well as exploring psychosocial aspects and the impact of SCI levels. Additionally, culturally appropriate and evidence-based interventions should be developed and evaluated to improve the psychosocial outcomes of SCI patients in Iran.

In conclusion, this narrative literature review provides a comprehensive overview of various aspects of SCI in Iranian patients. The review identifies several challenges and difficulties that SCI patients face in their daily lives, as well as some protective factors that can enhance their well-being. Additionally, the review highlights gaps and limitations in the current literature and suggests directions for future research. The findings of this review can inform policymakers, healthcare providers, researchers, and resources for SCI patients in Iran. The review concludes by recommending more in-depth and comprehensive studies on SCI patients in Iran, investigating the impact of different interventions and support systems, and addressing the research gaps identified in the current literature. By addressing these recommendations, future studies and interventions can contribute to a better understanding of SCI patients’ needs and challenges in Iran and inform the development of effective policies and support systems to improve their quality of life and overall well-being.

The limitation of our study is that we do not consider the independence of patients with SCI. The issue of independence is an important consideration for patients with SCI. Therefore, we suggest that future studies in Iran consider including a focus on the independence of patients with SCI. This could include studies on the effectiveness of rehabilitation programs, assistive devices, and other interventions aimed at enhancing the independence of patients with SCI. By addressing this issue, we can further contribute to the improvement of survival and quality of life for patients with SCI in Iran.
